# Supplementary material for: Development of standard indicators to assess use of electronic health record systems implemented in low-and medium-income countries
Source: PLoS One. 2021 Jan 11;16(1):e0244917. doi: 10.1371/journal.pone.0244917 (PMC7799790; doi:10.1371/journal.pone.0244917)
Supplement: S1 Appendix — (PDF) [file pone.0244917.s001.pdf]

S1 Appendix. System usage indicator template

<table>
<tr><td colspan="5">Indicator Name</td></tr>
<tr><td>Description:</td><td colspan="4">Long name of the indicator</td></tr>
<tr><td>Numerator:</td><td colspan="2">Long name of the numerator</td><td colspan="2">Additional information about numerator definition</td></tr>
<tr><td>Denominator</td><td colspan="2">Long name of the denominator</td><td colspan="2">Additional information about denominator definition</td></tr>
<tr><td>How to use:</td><td colspan="4">How data are used to monitor the Metric</td></tr>
<tr><td>How to collect:</td><td colspan="4">How the data are collected (highlight data source, issues with double counting and important components of data collection that ensure data quality)</td></tr>
<tr><td>PEPFAR MER 2.0 Considerations</td><td colspan="4">Key considerations between metric and other routinely collected PEPFAR MER indicators.</td></tr>
<tr><td>Reporting level</td><td colspan="4">Reported at facility, community, central</td></tr>
<tr><td>How often to report:</td><td colspan="4">From the quick reference guide</td></tr>
<tr><td>How to review for data quality:</td><td colspan="4">How data quality is reviewed for the specific indicator</td></tr>
<tr><td>How to calculate annual total:</td><td colspan="4">From the quick reference guide</td></tr>
<tr><td rowspan="4">Data Elements (Components of indicator)</td><td rowspan="2">Numerator: Long name of the numerator</td><td>Disaggregate Groups</td><td>Disaggregates</td><td>Description of Disaggregate</td></tr>
<tr><td></td><td></td><td></td></tr>
<tr><td rowspan="2">Denominator (Optional) Long name of the denominator:</td><td>Disaggregate Groups</td><td>Disaggregates</td><td>Description of Disaggregate</td></tr>
<tr><td></td><td></td><td></td></tr>
</table>

NOTE: Template borrowed from the HIV MER 2.0 indicator template which implementations are already familiar with.[35]
