# Supplementary material for: Development of standard indicators to assess use of electronic health record systems implemented in low-and medium-income countries
Source: PLoS One. 2021 Jan 11;16(1):e0244917. doi: 10.1371/journal.pone.0244917 (PMC7799790; doi:10.1371/journal.pone.0244917)
Supplement: S2 Appendix — (PDF) [file pone.0244917.s002.pdf]

S2 Appendix. Indicator rating form

Participant ID________

Indicator Name										
Indicator SMART Survey		**Rating**								
	Specific	1	2	3	4	5				
	Measurable	1	2	3	4	5				
	Achievable	1	2	3	4	5				
	Relevant	1	2	3	4	5				
	Timebound	1	2	3	4	5				
Include indicator?	Yes	No								
Overall rating	1	2	3	4	5	6	7	8	9	10

Comments on the indicator

____
____
____
____
____
____
____
____
____
____
