# Supplementary material for: Development of standard indicators to assess use of electronic health record systems implemented in low-and medium-income countries
Source: PLoS One. 2021 Jan 11;16(1):e0244917. doi: 10.1371/journal.pone.0244917 (PMC7799790; doi:10.1371/journal.pone.0244917)
Supplement: S3 Appendix — (DOCX) [file pone.0244917.s003.docx]

**S3 Appendix. Monitoring, Evaluation and Reporting (MER v1.0): Electronic Health Record (EHR) system usage indicator reference guide**

**Monitoring, Evaluation and Reporting (MER v1.0):**

**Electronic Health Record (EHR) system usage**

**indicator reference guide**

**Mar-1-2020**

**Version 1**

**TABLE OF CONTENTS**

[1. OVERVIEW: 3](#_Toc36806850)

[A. INTRODUCTION: 3](#_Toc36806851)

[B. OVERALL APPROACH TO INDICATOR IDENTIFICATION 3](#_Toc36806852)

[2. STEP 1: INITIAL INDICATOR IDENTIFICATION: 3](#_Toc36806853)

[2A: PROPOSED EMR INDICATORS 4](#_Toc36806854)

[BASELINE DATA 4](#_Toc36806855)

[ROUTINE REPORTING INDICATORS 5](#_Toc36806856)

[3. HOW TO READ THE INDICATOR REFERENCE SHEET: 6](#_Toc36806857)

[4. DETAILED EHR INDICATORS: 7](#_Toc36806858)

# 1. Overview

## A. Introduction

Many health facilities in the developing world are increasingly implementing Electronic Health Record Systems (EHRs) with the goal of improving clinical practice, supporting efficient health reporting and improving quality of care provided. Countries like Rwanda, Uganda, Mozambique and Kenya have gone beyond isolated and pilot implementations to large-scale rollout of select EHRs at government-run facilities. Large scale implementations present unique challenges and countries are finding themselves in the unenviable position of tracking the status of each implementation especially in settings with large geographic distribution.

A core consideration for each implementation is how well the implementation is functioning, and what the level of usage is for the EHRs both in patient care and reporting. System use is considered to be an important measure in evaluating information system (IS) success. As related to EHRs implementations, a standardized approach to measuring EMRs system use across implementations will: (1) ensure that up-to-date status of every implementation is known, with opportunity for improvements done, (2) monitoring of performance across implementing partners, systems, regions and countries can be conducted, and (3) barriers and challenges to implementation can be identified.

To this end, a set of ‘Electronic Health Record (EHR) System Usage’ Indicators, will be developed with guidance and leadership by CDC Atlanta.

## B. Overall approach to indicator identification

The approach to selecting and validating indicators will involve a multi-step process including:

(1) Initial development of suggested metrics and indicators by the consultant in close collaboration with CDC.

(2) Nominal Group technique exercise with 10-15 stakeholders with diverse backgrounds to refine, update and add to list of indicators.

(3) Revision and prioritization of identified indicators.

(4) Larger group review of indicators with CDC leadership.

(5) Concurrent real-world testing of select indicators.

(6) Concurrent work to endorse indicators for national use, and by central CDC.

# 2. Step 1: Initial indicator identification

The initial set of indicators were identified through close collaboration with CDC Atlanta. Given that the emphasis was on developing indicators to evaluate actual status of EHR use at the implementation sites (and not to evaluate technical functionality within the EHRs themselves), the key indicators developed included the following domains: (1) System Use, (2) Data Quality, (3) Interoperability, and (4) Reporting. It was recognized that baseline EHR data were also needed if further analyses were to be done based on particular characteristics surrounding the EHR implementation (**Figure 1**). Particular emphasis was placed on developing indicators that were **S**pecific, **M**easurable, **A**chievable, **R**elevant and **T**ime bound (SMART) within settings with limited resources.


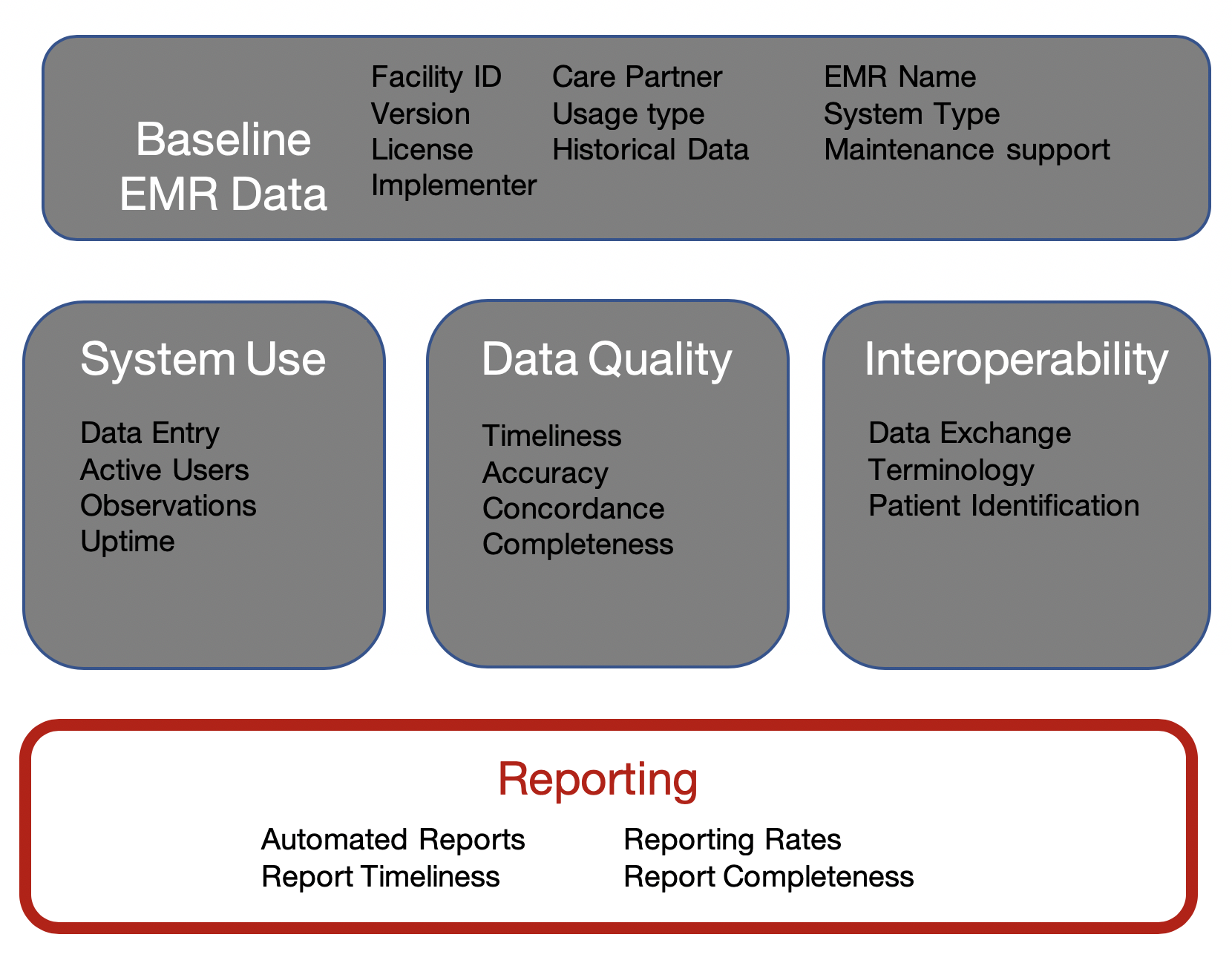


Figure 1: Infographic of key domains for EHRs Use indicators

## 2a: Proposed EHR indicators

### BASELINE DATA

It is proposed that a core set of information be made available and maintained on all EHRs implementations. This information will be important in helping to better understand the findings from the various indicators, especially when analysis is needed across various dimensions - such as by EHR system, by district, by implementation partner etc.

Proposed baseline data to be collected are outlined in Table 1:

**TABLE 1: PROPOSED BASELINE EHR DATA**

|  | **Domain** | **Indicator Name** | **Reporting Frequency** |
| --- | --- | --- | --- |
| 1 | Baseline Data | Facility Name or ID | At implementation & with every change |
| 2 | Baseline Data | Care Implementing Partner | At implementation & with every change |
| 3 | Baseline Data | Digital System Name | At implementation & with every change |
| 4 | Baseline Data | Implemented System Version | At implementation & with every upgrade |
| 5 | Baseline Data | Usage mechanism | At implementation & with every change |
| 6 | Baseline Data | System Type | At implementation & with every change |
| 7 | Baseline Data | System implementing partner | At implementation & with every change |
| 8 | Baseline Data | License | At implementation & with every change |
| 9 | Baseline Data | System maintenance support | At implementation & with every change |
| 10 | Baseline Data | System enhancement support | At implementation & with every change |
| 11 | Baseline Data | Historical Data | At implementation & with every change |

### ROUTINE REPORTING INDICATORS

The following initial set of indicators were identified for consideration for the initial set of indicators. It should be noted that the frequency for collecting the indicators is subject to discussion and revisions.

| **#** | **Domain** | **Indicator** | **Description** | **Proposed Frequency** |
| --- | --- | --- | --- | --- |
| 1 | System Use | Data Entry Statistics | Percentage of clinical encounters or visits for patients with HIV (both pre-ART and ART patients) within last quarter that were captured using the Electronic Record System. | Quarterly |
| 2 | System Use | Staff System Use | Percentage of health workers working on HIV-related activities (i.e. prevention, treatment and other HIV support for which data is stored in the EMR), who used the EMR system for patient care according to plan during the reporting period. | Quarterly |
| 3 | System Use | Observations | Number of new HIV-related clinical data elements recorded for patients in the EMR during the reporting period. | Quarterly |
| 4 | System Use | Uptime | % of time system is up when needed during the critical business function of taking care of patients. | Quarterly |
| 5 | Data Quality | Clinical Data Timeliness | Percentage of clinical provider encounters that are entered into the electronic record system within the agreed time period. | Quarterly |
| 6 | Data Quality | EMR Data Concordance | The extent to which data contained in paper forms compare to those transcribed into the EMR. Data elements where both data sources have missing values are considered to be concordant. (*Indicator only applies for facility that use paper records with the data transcribed into the EMR).* | Quarterly |
| 7 | Data Quality | EMR Data Completeness | The extent to which all required data elements for a patient are contained within the EMR. | Quarterly |
| 8 | Interoperability | Data Exchange | The extent to which the EMR can automatically exchange all required data with identified external applications. | Quarterly |
| 9 | Interoperability | Standardized Terminology | The proportion of key terminologies that are mapped to standard terminology services or use a nationally-endorsed concept dictionary. | Quarterly |
| 10 | Interoperability | Patient Identification | Extent of use of a nationally-accepted patient identification methods. | Quarterly |
| 11 | Reporting | Automatic Reports | The proportion of expected reports to the national level that are automatically generated and transmitted to the national reporting system. | Quarterly |
| 12 | Reporting | Reporting Rate | Percentage of expected EMR-based reports that are successfully submitted to the national reporting system (i.e. the reporting rate). | Quarterly |
| 13 | Reporting | Reporting Timeliness | Percentage of expected EMR-based reports that are submitted on time. | Quarterly |
| 14 | Reporting | Reporting Completeness | Percentage of expected EMR-based reports that are successfully submitted to the national reporting system (i.e. the reporting rate). | Quarterly |

# 3. How to read the indicator reference sheet

The EHR indicator reference sheet is modelled after the reference sheet for the HIV MER, given that implementing partners are familiar with these indicators.

All indicators are in a standard format in order to easily understand them. Please use this layout as a reference guide to understand how to read the reference sheets.

| Indicator Name | | | | | |
| --- | --- | --- | --- | --- | --- |
| **Description:** | *Long name of the indicator* | | | | |
| **Numerator:** | *Long name of the numerator* | | *Additional information about numerator*  *definition* | | |
| **Denominator** | *Long name of the denominator* | | *Additional information about denominator*  *definition* | | |
| **How to use:** | *How is data used to monitor the Metric* | | | | |
| **How to collect:** | *How is the data collected (highlight data source, issues with double counting and important components of data collection that ensure data quality)* | | | | |
| **PEPFAR MER 2.0 Considerations** | *Key considerations between metric and other routinely collected PEPFAR MER indicators.* | | | | |
| **Reporting level** | *Reported at facility, community, central* | | | | |
| **How often to report:** | *From the quick reference guide* | | | | |
| **How to review for data quality:** | *How is data quality reviewed for the specific indicator.* | | | | |
| **How to calculate annual total:** | *From the quick reference guide* | | | | |
| **Data Elements (Components of indicator)** | **Numerator**:  *Long name of*  *the numerator* | **Disaggregate**  **Groups** | | **Disaggregates** | **Description of**  **Disaggregate** |
|  |  |  | |  |  |
|  | **Denominator *(Optional)*** *Long name of the*  *denominator*: | **Disaggregate**  **Groups** | | **Disaggregates** | **Description of**  **Disaggregate** |
|  |  |  | |  |  |

# 4. Detailed EHR indicators:

| **System Use** |
| --- |
| **Indicator Reference Sheets** |
| MER Ver 1 |

| **#** | **Domain** | **Indicator** | **Description** | **Proposed Frequency** |
| --- | --- | --- | --- | --- |
| 1 | System Use | Data Entry Statistics | Percentage of clinical encounters or visits for patients with HIV (both pre-ART and ART patients) within last quarter that were captured using the Electronic Record System. | Quarterly |
| 2 | System Use | Staff System Use | Percentage of health workers working on HIV-related activities (i.e. prevention, treatment and other HIV support for which data is stored in the EMR), who used the EMR system for patient care according to plan during the reporting period. | Quarterly |
| 3 | System Use | Observations | Number of new HIV-related clinical data elements recorded for patients in the EMR during the reporting period. | Quarterly |
| 4 | System Use | Uptime | % of time system is up when needed during the critical business function of taking care of patients. | Quarterly |

| **INDICATOR NAME: DATA ENTRY STATISTICS** | | | | | |
| --- | --- | --- | --- | --- | --- |
| **Description:** | Percentage of clinical encounters or visits for patients with HIV within last quarter that were captured using the Electronic Record System. | | | | |
| **Numerator:** | Number of patient visits captured in the EMRs during the reporting period. | | If there is more than one visit by patient during the quarter, report all visits in the numerator. | | |
| **Denominator** | Total number of unique patient visits or clinical encounters that are occurred during the reporting period. | | Some of these visits could be captured via registers or paper-based encounter forms. All have to be counted. | | |
| **How to use:** | This indicator is used to monitor completeness of clinical record capture into the electronic system during the reporting period. Essentially, how well routine clinical data is being captured through the electronic record system. | | | | |
| **How to collect:** | This indicator should be collected from the clinical source to assure unduplicated patient clinic visit counting. Information should come from all sources that capture clinic visits including from electronic system (EMR) or registers. If standard registers or EMR queries do not contain all the required information, individual patient files should be reviewed to determine if a visit occurred. The numerator is often a query within the EMR to determine number of unique relevant clinical encounters entered during the project period, excluding updates, or encounters not relevant to be counted.  NOTE: Clinic visits of interest are initial or return clinic visits with providers for pre-ART and ART patients. Numbers should be reported for all patients, including both pediatric and adult patients, and those coming for PMTCT. Drug pick-up visits, counseling visits, or opportunistic infection visits (e.g. to the TB clinic) are not included. | | | | |
| **PEPFAR MER Considerations** | N/A. | | | | |
| **Reporting level** | Site level | | | | |
| **How often to report:** | Quarterly | | | | |
| **How to review for data quality:** | Numerator ≤ Denominator. Number of patient visit records captured in the system during the reporting period equal or less than Total number of patient visit records captured through all modalities. | | | | |
| **How to calculate annual total:** | Annual rate will be the average of quarterly reporting percentages. | | | | |
| **Data Elements (Components of indicator)** | ***Numerator (Required)****:* Number of adults and pediatric HIV patients (both pre-ART and on ART) with documented clinic visit record within the Electronic record systems within the reporting quarter | **Disaggregate**  **Groups**  **Patient Category**  Adult  Pediatric  PMTCT  **Data Entry Type**  Point of care  Retrospective  **(Required)** | | **Disaggregates** | **Description of**  **Disaggregate** |
|  |  |  | |  |  |
|  | **Denominator *(Required)***  Number of adults and pediatric HIV patients (both pre-ART and on ART) with documented clinic visit record from anywhere in the system, including electronic records, paper charts and registry. | **Disaggregate**  **Groups** | | **Disaggregates** | **Description of**  **Disaggregate** |
|  |  | **Disaggregate**  **Groups** | |  |  |

| **INDICATOR NAME: STAFF SYSTEM USE** | | | | | |
| --- | --- | --- | --- | --- | --- |
| **Description:** | Percentage of staff members who used the EMR system during the reporting period. | | | | |
| **Numerator:** | Number of active users of the system during reporting period. | | Individuals with privileges to use the system are expected to have logged into the system during the reporting period for a time duration that is deemed meaningful by the country. | | |
| **Denominator** | Total number of staff members with privileges to use the system. | | This includes all staff providers given privileges within the system. | | |
| **How to use:** | This indicator is used to monitor how well people with privileges to use the system are actually using it. | | | | |
| **How to collect:** | The country’s standard operating procedures should define what meaningful duration of access to the EMR is. As an example, a country could define it as the total length of time an individual is logged into system (e.g. at least 2 hours during the reporting period), or the length of a session (e.g. they must be logged in for at least 10 minutes in a session during the reporting period).  This indicator will be derived from EMR queries of log data. The numerator is often a query within the EMR to determine if a particular staff member accessed the EMR to meet the criteria defined by the country. The denominator is also derived from the EMR as the total number of providers with access to the system. | | | | |
| **PEPFAR MER Considerations** | None. While HRH_STAFF and HRH_CURR under PEPFAR MER 2.0 could seem relevant, this current indicator is for any full or part-time provider offering HIV care services for which data is entered in to the EMR. | | | | |
| **Reporting level** | Facility, district, national. | | | | |
| **How often to report:** | Quarterly | | | | |
| **How to review for data quality:** | Numerator ≤ Denominator. There is a risk of artificially inflated access to meet indicator goal. Getting distribution of access by provider could help highlight this discrepancy. | | | | |
| **How to calculate annual total:** | Annual rate will be the average of quarterly reporting percentages. | | | | |
| **Data Elements (Components of indicator)** | ***Numerator (Required)****:*  Number of active users of the system during reporting period. | **Disaggregate**  **Groups**  User Category  Access Type  -Clinical data entry  -Patient chart review  - Reporting  **(Required)** | | **Disaggregates** | **Description of**  **Disaggregate**  User Category defines the type of users who accessed the system - e.g. providers, managers, data entry clerks.  Access Type: Access type defines what the access was for. Given a sense of what the access is used for. |
|  |  |  | |  |  |
|  | **Denominator *(Required)***  Total number of staff members with privileges to use the system. | **Disaggregate**  **Groups** | | **Disaggregates** | **Description of**  **Disaggregate** |
|  |  | **Disaggregate**  **Groups** | |  |  |

**Revision after NGT Exercise:**

- Team suggested that the original indicator was too difficult to actualize, and proposed simplifying it.
- Changed from health care worker access to all provider access.
- Changed from proportion of patient records accessed during visit, to simply provider access to the system as this is easier to measure.
- The team suggested that the indicator should be changed to ‘Number of Active Users of the System during Reporting Period’. We changed this to proportion, as we want to capture those who are not using the system - to help in understanding why.
- Meaningful use concept introduced - though this has to be defined by country. It could be total duration of time for system use during period, or length of session.

| **INDICATOR NAME: OBSERVATIONS** | | | | | |
| --- | --- | --- | --- | --- | --- |
| **Description:** | Number of mandatory HIV-related clinical data elements recorded for patients in the EMR during the reporting period. | | | | |
| **Numerator:** | Number of mandatory HIV-related clinical data elements recorded for patients in the EMR during the reporting period. | | The numerator is the sum of all new clinical data captured within the EMR from site. | | |
| **Denominator** | N/A | |  | | |
| **How to use:** | This indicator is important to measure the volume of HIV-related clinical data being captured into the EMR from the various sites. Country should define which types of data elements need to be recorded (e.g. those transmitted to the national data warehouse) and report on these numbers. It can be matched against the national data warehouse data to see if required observational data is being sent to the data warehouse. | | | | |
| **How to collect:** | This data should be collected as a query from within the EMR. It should capture the most current unique patient-related clinical data elements for HIV-care captured within the system. Updated data should only be counted once, but updates to data from previous reporting periods should not be counted. Voided or deleted data should not be counted. | | | | |
| **PEPFAR MER Considerations** | None. | | | | |
| **Reporting level** | Site level, District, Country | | | | |
| **How often to report:** | Quarterly | | | | |
| **How to review for data quality:** | Query used in this indicator should be well-tested. Ensure that data counted does not include data for non-HIV related clinic visits - especially for implementations that store data within the system for none HIV care. Update data from previous reports should there be any changes. | | | | |
| **How to calculate annual total:** | Annual rate will be the average of quarterly reporting percentages. | | | | |
| **Data Elements (Components of indicator)** | ***Numerator (Required)****:*  Number of new HIV-related clinical data elements recorded for patients in the EMR during the reporting period. | **Disaggregate**  **Groups**  **Data Type**  Patient Demographics  Clinic visit data  Laboratory  Pharmacy  **(optional)** | | **Disaggregates** | **Description of**  **Disaggregate**  Patient Demographics - new registration data for patients  Clinic visit data - data captured on clinic encounter forms.  Laboratory - data from laboratory capture forms or Laboratory information systems.  Pharmacy - data from pharmacy forms or pharmacy information system. |
|  |  |  | |  |  |
|  | **Denominator *(Required)***  N/A | **Disaggregate**  **Groups** | | **Disaggregates** | **Description of**  **Disaggregate** |
|  |  | **Disaggregate**  **Groups** | |  |  |

**Revision after NGT Exercise:**

- The groups suggested that we focus only on mandatory data elements required for collection - which will be defined by country. The initial indicator focussed on all data recorded in the EMRs.

| **INDICATOR NAME: UPTIME** | | | | | |
| --- | --- | --- | --- | --- | --- |
| **Description:** | % of time system is up when needed during the critical business function of taking care of patients. | | | | |
| **Numerator:** | Agreed Service Time (AST) minus Downtime (DT) | | Country SOP should define the amount of time that the EMR should be available over the reporting period (AST) - and this could be by facility (as some facility work on different days). Downtime (DT) or outage is time when system is not available for use during the agreed service time. DT can be either scheduled or unscheduled - but is relevant as long as it occurs during agreed service time. | | |
| **Denominator** | Agreed Service Time (AST) - the amount of time that the EMR should be available over the reporting period. | |  | | |
| **How to use:** | This indicator is important in measuring the availability of the system for use when it is needed. Low uptime rates means that the EMR is not available for use when needed either for patient care or reporting. | | | | |
| **How to collect:** | This data should ideally be collected from system logs that show whether the system is up and running. Ideally, EMRs should have a way of automatically measuring uptime, downtime and system off times. The key measure here is whether patient care users can have access to patient records and can record new patient information through the EMR.  Ideally, this data should be collected continually by facilities. | | | | |
| **PEPFAR MER Considerations** | None. | | | | |
| **Reporting level** | Site level, District, Country | | | | |
| **How often to report:** | Monthly | | | | |
| **How to review for data quality:** | Downtime cannot be more than agreed service time. Recorded times should not be outside window of agreed service time. | | | | |
| **How to calculate annual total:** | Annual rate will be the average of reported percentages. | | | | |
| **Data Elements (Components of indicator)** | ***Numerator (Required)****:*  Agreed Service Time (AST) minus Downtime (DT) | **Disaggregate**  **Groups**  **Downtime Reason**  Planned downtime  Unplanned downtime   - System problem - Power problem   By facility type  By EMRs system  **(optional)** | | **Disaggregates** | **Description of**  **Disaggregate**  Planned downtime - this is scheduled downtime usually for system maintenance and upgrades. This ideally should be done outside Agreed Service time(AST), but if it occurs during AST, it should be noted.  Unplanned downtime  This is system downtime that occurs for unforeseen reasons. General system software problems and power problems can be a cause of this. |
|  |  |  | |  |  |
|  | **Denominator *(Required)***  Agreed service time | **Disaggregate**  **Groups** | | **Disaggregates** | **Description of**  **Disaggregate** |
|  |  | **Disaggregate**  **Groups** | |  |  |

**Revision after NGT Exercise:**

- Previous denominator did not have a definition of Agreed Service Time (AST). This was added.
- Participants stated that the AST will likely vary by facility.
- The team felt strongly that we should avoid anything that requires self-reporting. As such, this element has been removed, and indicator will be derived ideally from just EMRs queries.
- Add disaggregation by facility type, and by EMRs system type.

| **Data Quality** |
| --- |
| **Indicator Reference Sheets** |
| MER Ver 1 |

| **#** | **Domain** | **Indicator** | **Description** | **Proposed Frequency** |
| --- | --- | --- | --- | --- |
| 5 | Data Quality | Clinical Data Timeliness | Percentage of clinical provider encounters that are entered into the electronic record system within the agreed time period. | Quarterly |
| 6 | Data Quality | EMR Variable Concordance | The extent to which data contained in paper forms compare to those transcribed into the EMR. Data elements where both data sources have missing values are considered to be concordant. (*Indicator only applies for facility that use paper records with the data transcribed into the EMR).* | Quarterly |
| 7 | Data Quality | EMR Variable Completeness | The extent to which all required data elements for a patient are contained within the EMR. | Quarterly |

| **INDICATOR NAME: CLINICAL DATA TIMELINESS** | | | | | |
| --- | --- | --- | --- | --- | --- |
| **Description:** | Percentage of clinical provider encounters that are entered into the electronic record system within the agreed time period. | | | | |
| **Numerator:** | Number of clinical provider encounters that are fully entered into the Electronic record system within the agreed time during the reporting period. | | Country SOP should define the length of acceptable time duration within which a clinical encounter should be entered into the electronic record system. This can be within hours, or within days, based on what the country deems important. Acceptable time duration should be guided by when clinical data would find use either for subsequent patient reviews and care (e.g. data lookup), or for reporting.  This indicator only applies to clinical provider encounter data - i.e. a patient visit with a provider for which clinical data is captured within accepted forms or formats in the EMRS. It does not include data (such as laboratory studies) that come from other systems, as lag time is affected by multiple other factors. | | |
| **Denominator** | Total Number of Clinical Encounters | | This should include all encounters that were entered within the system and those that are not entered. As an example, if a facility does not enter their data into the EMRS, their Clinical Data Timeliness will be 0 percent. If a facility only enters half their data into the EMRS but do these within the acceptable time, their Clinical Data Timeliness will still be 50%. | | |
| **How to use:** | This indicator is important in determining the availability and accessibility of data for clinical, administrative and reporting decision-making. | | | | |
| **How to collect:** | The total number of encounters (Denominator) during the reporting period should be determined by simply counting the number of visits, similar to the denominator in ‘DATA ENTRY STATISTICS’.  The numerator should ideally be generated from a query within the EMRS, which compares the DATE-TIME for the Clinical Encounter and the DATE-TIME for when the data was entered into the system (this signifies when the data was actually marked as saved within the system). The time difference is then compared with the length of acceptable time duration by the country to see whether the encounter was entered in a timely fashion.  It is recognized that the exact time for some encounters might not be available, and in these situations (which will be the case in most countries), the timeliness indicator should be calculated based on dates.  Ideally, facilities should track the denominator continually. The numerator can be calculate with a query to the EMRS database. | | | | |
| **PEPFAR MER Considerations** | None. | | | | |
| **Reporting level** | Site level, District, Country | | | | |
| **How often to report:** | Monthly | | | | |
| **How to review for data quality:** | Queries applied should be vetted to avoid counting the same clinical encounter multiple times - given the situation where data is erroneously entered multiple times in the EMRs. The clinical visits to count should only be those for which there is a requirement to enter the encounter information into the EMRS. There is need to watch out errors in reported timeliness especially during data reconstruction. | | | | |
| **How to calculate annual total:** | Annual rate will be the average of reported percentages. | | | | |
| **Data Elements (Components of indicator)** | ***Numerator (Required)****:*  Clinical encounters entered into the system in a timely fashion. | **Disaggregate**  **Groups**  **By Role:**  Provider  Data entry clerk  Encounter type  **By Type of System:**  Point of Care  Retrospective entry  **(Required)** | | **Disaggregates** | **Description of**  **Disaggregate**   - Provider - identify if there is a difference in data entry lag time by clinical provider who saw patients and sometimes entered the data into system. - Data entry clerk - in settings where data entry clerks are used to enter EMRS data retrospectively, it would be good to know if lag time varies by clerk. - Encounter type - given the varying length and complexity of various types of clinical visits and forms, there might be significant differences in how well data for particular encounters are entered into the system. |
|  |  |  | |  |  |
|  | **Denominator *(Required)***  Total number of clinical encounters | **Disaggregate**  **Groups** | | **Disaggregates** | **Description of**  **Disaggregate** |
|  |  | **Disaggregate**  **Groups** | |  |  |

**Revision after NGT Exercise:**

- Add disaggregation by type of system - e.g. point of care, retrospective etc.
- In ‘How to Review Data Quality’, add the risk in indicator reporting errors associated with data reconstruction (might make timeliness result off).

| **INDICATOR NAME: EMR VARIABLE CONCORDANCE** | | | | | |
| --- | --- | --- | --- | --- | --- |
| **Description:** | The extent to which data contained in paper forms compare to those transcribed into the EMR. Data elements where both data sources have missing values are considered to be concordant. (*Indicator only applies for facility that use paper records with the data transcribed into the EMR).* | | | | |
| **Numerator:** | Number of data elements with concordant values on paper form and EMR. | | The country SOP should define 10 key data elements related to HIV care and treatment that are entered from paper into the EMR that will be used to assess data concordance. These should be data elements relevant to 90-90-90.  Country SOP should also define how records should be sampled. It could be 5 or 10% of all records entered into the EMR, or a specific number (e.g. 100). This number can vary based on where the country is on the maturity model - e.g. countries just starting off or facilities that have done data reconstruction should likely review more records.  *(Example data elements: Patient ID, sex, date of birth, enrollment date, enrollment program, entry point, last visit date, next visit date, number of clinic visits, first CD4 count, last CD4 count, first WHO stage, last WHO stage, last co-trimoxazole date, ART start date, ART regimen, weight, transfer in date, transfer out date, and date of death).* | | |
| **Denominator** | Total Number of data elements. | |  | | |
| **How to use:** | This indicator is important in determining the accuracy of transcription of paper-based patient data into the EMR. | | | | |
| **How to collect:** | The assessment team should enumerate all clients who have ever received HIV care and treatment at the facility and whose data is entered into the EMR. They should randomly sample approximately a proportion of client records for review. Teams abstract values for 10 data elements related to HIV care and treatment services from both paper records and EMR. The assessment compares values between paper records and EMR.  For this exercise an excel-based tool will be provided into which data from the paper forms and EMR are abstracted. This tool automatically generates graphs showing the completeness and concordance for all 20 data elements. For each record reviewed, a concordance score equal to the number of data elements with concordant values on the paper form and EMR is generated. Data elements where both data sources are missing values are considered to be concordant. A score of 20 indicates that all data elements for that record were concordant, while 0 indicates that no data elements were concordant.  *Reference: Muthee V, Bochner AF, Osterman A, Liku N, Akhwale W, Kwach J, et al. (2018) The impact of routine data quality assessments on electronic medical record data quality in Kenya. PLoS ONE 13 (4): e0195362. https://doi.org/11371/journal.* | | | | |
| **PEPFAR MER Considerations** | None. | | | | |
| **Reporting level** | Site level, District, Country | | | | |
| **How often to report:** | Quarterly | | | | |
| **How to review for data quality:** | Double-data entry can be instituted within the excel-based tool to ensure accuracy of the data entered into the tool. Where there are discrepancies in entered data into the excel evaluation tool, the numbers should be corrected. | | | | |
| **How to calculate annual total:** | Annual rate will be the average of reported percentages. | | | | |
| **Data Elements (Components of indicator)** | ***Numerator (Required)****:*  Number of data elements with concordant values on paper form and EMR. | **Disaggregate**  **Groups**  By variable  **(Required)** | | **Disaggregates** | **Description of**  **Disaggregate** |
|  |  |  | |  |  |
|  | **Denominator *(Required)***  Total number of data elements assessed | **Disaggregate**  **Groups** | | **Disaggregates** | **Description of**  **Disaggregate** |
|  |  | **Disaggregate**  **Groups** | |  |  |

**Revision after NGT Exercise:**

- Change name from ‘EMR Data Concordance’ to ‘EMR Variable Concordance’. This will ensure that there is no confusion with ‘Report Concordance’
- Variables should be picked from those that are key for HIV care, and conform to 90-90-90. It was noted that some countries had these key variables already identified, while others did not. In either case, a core number should be chosen.
- The NGT participants recommended reducing number of variables to be assessed from 25 to 10 to make it more achievable.
- The team recommended that the frequency should be increased from Yearly to Quarterly.
- Not concluded was the number of records to review. 100 records was selected based on the study by Muthee et al. However, an approach could be to take a percentage (e.g. 5-10%) of randomly selected records for review. It was also suggested that based on the stage of the country (according to maturity model) countries lower on the model should consider doing more indicators. Countries also doing data reconstruction should consider doing more indicators.
- Disaggregation by variable.

| **INDICATOR NAME: EMR VARIABLE COMPLETENESS** | | | | | |
| --- | --- | --- | --- | --- | --- |
| **Description:** | The extent to which all required data elements for a patient are contained within the EMR. | | | | |
| **Numerator:** | Number of required data elements that are contained within the EMR. | | The country SOP should define 10 key data elements related to HIV care and treatment that are entered from paper into the EMR that will be used to assess data concordance. These should be data elements relevant to 90-90-90. Ideally, the same data elements should be used for the ‘EMR Variable Concordance’ and ‘EMR Variable Completeness’ Indicators.  Country SOP should also define how records should be sampled. It could be 5 or 10% of all records entered into the EMR, or a specific number (e.g. 100). This number can vary based on where the country is on the maturity model - e.g. countries just starting off or facilities that have done data reconstruction should likely review more records.  *(Example data elements: Patient ID, sex, date of birth, enrollment date, enrollment program, first CD4 count, first WHO stage, last WHO stage, last co-trimoxazole date, ART start date, ART regimen, weight, transfer in date, transfer out date, and date of death).* | | |
| **Denominator** | Total number of required data elements. | |  | | |
| **How to use:** | This indicator is important in determining the completeness of data contained within the EMR, hence ensuring relevance and usefulness of the EMR data for care and reporting. | | | | |
| **How to collect:** | This data should be collected as a query from within the EMR. It should determine for each patient record within the system whether the required data elements exist. Voided or deleted data should not be counted. | | | | |
| **PEPFAR MER Considerations** | None. | | | | |
| **Reporting level** | Site level, District, Country | | | | |
| **How often to report:** | Quarterly | | | | |
| **How to review for data quality:** | Query should be vetted periodically to ensure that it accurately calculates completeness. | | | | |
| **How to calculate annual total:** | Annual rate will be the average of reported percentages. | | | | |
| **Data Elements (Components of indicator)** | ***Numerator (Required)****:*  Number of mandatory data elements that are contained within the EMR. | **Disaggregate**  **Groups**  Variable | | **Disaggregates** | **Description of**  **Disaggregate** |
|  |  |  | |  |  |
|  | **Denominator *(Required)***  Total number of data elements assessed | **Disaggregate**  **Groups** | | **Disaggregates** | **Description of**  **Disaggregate** |
|  |  | **Disaggregate**  **Groups** | |  |  |

**Revision After NGT Exercise:**

- Change title to ‘EMR variable completeness’ from ‘EMR Data Completeness’
- The data elements selected for Completeness should be the same as ‘Concordance’.
- Disaggregation should be done by data variable.

| **INTEROPERABILITY** |
| --- |
| **Indicator Reference Sheets** |
| MER Ver1 |

| **#** | **Domain** | **Indicator** | **Description** | **Proposed Frequency** |
| --- | --- | --- | --- | --- |
| 8 | Interoperability | Data Exchange | The extent to which the EMR can automatically exchange all required data with identified external applications. | Quarterly |
| 9 | Interoperability | Standardized Terminology | The proportion of key terminologies that are mapped to standard terminology services or use a nationally-endorsed concept dictionary. | Quarterly |
| 10 | Interoperability | Patient Identification | Extent of use of a nationally-accepted patient identification methods. | Quarterly |

| **INDICATOR NAME: DATA EXCHANGE** | | | | | |
| --- | --- | --- | --- | --- | --- |
| **Description:** | Percentage of specified systems with which the EMR can automatically exchange all required data with. | | | | |
| **Numerator:** | Number of required systems that exchanged data automatically with the EMR during the reporting period. | | The country SOP should define the number of systems with which the EMR should be able to automatically share data to and receive data from.  The SOP should also define what level of data exchange meets a good threshold for this indicator. It should be recognized that frequency of data exchange will vary by system (e.g. data to reporting systems might only be exchanged monthly or quarterly)  *(Examples include: Laboratory information systems, pharmacy information systems, mobile health applications, data aggregation systems such as DHIS2)* | | |
| **Denominator** | Total number of expected systems with which the EMR should be able to exchange data. | |  | | |
| **How to use:** | This indicator is ensuring that the EMR is able to exchange data automatically with key external systems identified by the country. | | | | |
| **How to collect:** | This data should be collected as a query from within the EMR. This query should be based on actual stored data or data logs, and should be implemented according to the SOP threshold by country. For each system where data exchange is expected, a calculation will be done as to whether the levels of data exchange met the thresholds set by the country SOP. Total number of systems meeting this threshold will be ones included in this indicator. | | | | |
| **PEPFAR MER Considerations** | None. | | | | |
| **Reporting level** | Site level, District, Country | | | | |
| **How often to report:** | Quarterly | | | | |
| **How to review for data quality:** | Query should be vetted periodically to ensure that it accurately calculates data exchange. | | | | |
| **How to calculate annual total:** | Annual rate will be the average of reported numbers. | | | | |
| **Data Elements (Components of indicator)** | ***Numerator (Required)****:*  Number of required systems that exchanged data automatically with the EMR during the reporting period. | **Disaggregate**  **Groups**  By system. | | **Disaggregates** | **Description of**  **Disaggregate**  This will list all systems that were able to exchange data with the EMR, and the systems that were not able to exchange data with the EMR during the reporting period. |
|  |  |  | |  |  |
|  | **Denominator *(Required)***  Total number of expected systems with which the EMR should be able to exchange data. | **Disaggregate**  **Groups** | | **Disaggregates** | **Description of**  **Disaggregate** |
|  |  | **Disaggregate**  **Groups** | |  |  |

**Revision after NGT Exercise:**

- Change the indicator from an absolute number to a percentage
- Do not just focus on external systems, include all systems where data exchange is expected.
- Change the wording in Description from ‘Extent to which data exchanged’ to ‘Percentage of specified systems’.
- Provide adequate time for exchange to occur - as an example, reporting systems might only need to exchange data with EMRS during reporting period e.g. quarterly.

| **INDICATOR NAME: STANDARDIZED TERMINOLOGIES** | | | | | |
| --- | --- | --- | --- | --- | --- |
| **Description:** | The proportion of key terminologies that are mapped to standard terminology services or use a nationally-endorsed concept dictionary. | | | | |
| **Numerator:** | Number of key concept terms that are mapped to standard terminology services or use a nationally-endorsed concept dictionary. | | The country SOP should define key concept terms that need to be mapped to standard terminology services or for which systems should use a nationally-endorsed concept dictionary. | | |
| **Denominator** | Total number of key concept terms that should be mapped. | |  | | |
| **How to use:** | This indicator is important in determining how well EMR systems use standardized terminology systems (e.g. ICD-10) or a nationally-endorsed concept dictionary. This allows for semantic interoperability between systems. | | | | |
| **How to collect:** | This data should be provided by the EMR implementing partner for their system. | | | | |
| **PEPFAR MER Considerations** | None. | | | | |
| **Reporting level** | Country | | | | |
| **How often to report:** | Yearly | | | | |
| **How to review for data quality:** | There needs to be a human review on whether the required concept terms are mapped, and their accuracy. | | | | |
| **How to calculate annual total:** | This indicator will be reported once. | | | | |
| **Data Elements (Components of indicator)** | ***Numerator (Required)****:*  Number of concept terms that are mapped to standard terminology services or use a nationally-endorsed concept dictionary. | **Disaggregate**  **Groups**    **(Required)** | | **Disaggregates** | **Description of**  **Disaggregate** |
|  |  |  | |  |  |
|  | **Denominator *(Required)***  Total number of data elements assessed | **Disaggregate**  **Groups** | | **Disaggregates** | **Description of**  **Disaggregate** |
|  |  | **Disaggregate**  **Groups** | |  |  |

**Revision after NGT Exercise:**

- Change to ‘key concept terms’
- There were discussions on whether this was a functionality indicator and not a use indicator.

| **INDICATOR NAME: PATIENT IDENTIFICATION** | | | | | |
| --- | --- | --- | --- | --- | --- |
| **Description:** | Use of a nationally-accepted patient identification method. | | | | |
| **Numerator:** | Number of patients with required nationally-endorsed identifiers entered as part of their record. | | The country SOP should define key REQUIRED individual identifiers that can be used for patient records.  Ideally, this should be one unique patient identifier, but in settings where a nationally-endorsed unique identifier does not exist, the required endorsed identifiers should be used. | | |
| **Denominator** | Total number of active patients within the EMR. | |  | | |
| **How to use:** | This indicator is used to ensure that nationally-endorsed identifiers are used for patients. | | | | |
| **How to collect:** | This data should be collected as a query from within the EMR. This query should be based on actual stored data, and should be implemented according to the SOP by the country. The goal is to identify how well facilities are recording the required patient identifiers.  The query will be done on each patient, and then patients meeting criteria for having the needed identifiers will be in the numerator. Total number of patients in the EMR will be the denominator.  The query will be re-calculated every time, as identifiers can be changed between reporting periods. | | | | |
| **PEPFAR MER Considerations** | None. | | | | |
| **Reporting level** | Country | | | | |
| **How often to report:** | Quarterly. | | | | |
| **How to review for data quality:** | EMR systems should have features to validate each identifiers - as such, this indicator will simply look at the existence of identifiers, and presume that if identifiers are recorded in the system, they will be validated.  The query will be re-calculated every time, as identifiers can be changed between reporting periods. It should not include deleted or voided values.  The indicator involves unique active (not dead or transferred out) patients. | | | | |
| **How to calculate annual total:** | Annual rate will be the average of reported percentages. | | | | |
| **Data Elements (Components of indicator)** | ***Numerator (Required)****:*  Number of patients with nationally-endorsed identifiers entered as part of their record. | **Disaggregate**  **Groups**  Identifier type  **(optional)** | | **Disaggregates** | **Description of**  **Disaggregate**  This looks at how well different nationally-endorsed identifiers are being recorded in system. |
|  |  |  | |  |  |
|  | **Denominator *(Required)***  Total number of unique patients within the EMR. | **Disaggregate**  **Groups** | | **Disaggregates** | **Description of**  **Disaggregate** |
|  |  | **Disaggregate**  **Groups** | |  |  |

**Revision after NGT Exercise:**

- Add disaggregation by identifier type.
- Recognize that identifiers could include machine-generated unique identifiers.

| **REPORTING** |
| --- |
| **Indicator Reference Sheets** |
| MER VER 1 |

| **#** | **Domain** | **Indicator** | **Description** | **Proposed Frequency** |
| --- | --- | --- | --- | --- |
| 11 | Reporting | Automatic Reports | The proportion of expected reports to the national level that are automatically generated and transmitted to the national reporting system. | Quarterly |
| 12 | Reporting | Reporting Rate | Percentage of expected EMR-based reports that are successfully submitted to the national reporting system (i.e. the reporting rate). | Quarterly |
| 13 | Reporting | Reporting Timeliness | Percentage of expected EMR-based reports that are submitted on time. | Quarterly |
| 14 | Reporting | Reporting Completeness | Percentage of expected EMR-based reports that are successfully submitted to the national reporting system (i.e. the reporting rate). | Quarterly |
| 15 | Reporting | Reporting  Concordance | The extent to which data contained in paper-derived reports compare to report data derived from the EMR. | Quarterly |

| **INDICATOR NAME: AUTOMATIC REPORTS** | | | | | |
| --- | --- | --- | --- | --- | --- |
| **Description:** | The proportion of expected reports and sub-reports to the national level that are automatically generated and transmitted to the national reporting system. | | | | |
| **Numerator:** | Number of expected reports and sub-reports that are automatically generated and successfully transmitted from EMR to the national reporting system during reporting period. | | The country SOP should identify that required reports or sub-reports that need to be derived from data within the EMR system. | | |
| **Denominator** | Total number of required reports or sub-reports based on EMR data during reporting period. | |  | | |
| **How to use:** | This indicator is important in determining how well EMR systems are able to automatically generate and transmit reports to the national data reporting system. | | | | |
| **How to collect:** | Ideally, this should be an automated query from within the EMR that determines whether a report is automatically generated, and confirms that the report was successfully transmitted. | | | | |
| **PEPFAR MER Considerations** | None | | | | |
| **Reporting level** | Facility, District, Country | | | | |
| **How often to report:** | In line with PEPFAR reports | | | | |
| **How to review for data quality:** | It is presumed that EMRs will have verified the quality of the components within reports. Ideally, reported data should occasionally be confirmed within the national reporting system, such as DHIS2. | | | | |
| **How to calculate annual total:** | This will be an average of all proportions reported over the year. | | | | |
| **Data Elements (Components of indicator)** | ***Numerator (Required)****:*  Number of reports or sub-reports that are automatically generated and successfully transmitted from EMR to the national reporting system during reporting period. | **Disaggregate**  **Groups**  Report type  **(Required)** | | **Disaggregates** | **Description of**  **Disaggregate**  This will measure how well different reports are being automatically generated. |
|  |  |  | |  |  |
|  | **Denominator *(Required)***  Total number of required reports or sub-reports based on EMR data during reporting period. | **Disaggregate**  **Groups** | | **Disaggregates** | **Description of**  **Disaggregate** |
|  |  | **Disaggregate**  **Groups** | |  |  |

**Revision after NGT Exercise:**

- Comment was made that this indicator could stay under reports or move to interoperability.
- It was mentioned that most reports usually contain sub-reports, and that SOP for countries should be clear if they refer to reports or sub-reports.
- These reports/sub-reports should be based on data purely derived from the EMRs. I.e. reports that do not require other pieces of information.

| **INDICATOR NAME: REPORTING RATE** | | | | | |
| --- | --- | --- | --- | --- | --- |
| **Description:** | Percentage of expected EMR-based reports or sub-reports that are successfully submitted to the national reporting system (i.e. the reporting rate). | | | | |
| **Numerator:** | Number of EMR-based reports or sub-reports submitted | | Country SOP should define the reports or sub-reports that are generated from data contained in the EMR that have to have been submitted to the national reporting system.  The number of expected reports or sub-reports will likely vary by facility. | | |
| **Denominator** | Expected number of EMR-based reports or sub-reports. | | This indicator only applies to reports or sub-reports for data contained within the EMR. | | |
| **How to use:** | This indicator is important in determining the availability and accessibility of data for clinical, administrative and reporting decision-making. | | | | |
| **How to collect:** | This indicator is calculated directly for each facility from the data aggregation system, such as DHIS2 as these systems already contain these date.  Report rate is calculated based on a whether the reports have been submitted to the national reporting system.  **Percentage** reporting rate is calculated by:  (Number of actual reports submitted / Expected number of reports) * 100 | | | | |
| **PEPFAR MER Considerations** | None. | | | | |
| **Reporting level** | Site level, District, Country | | | | |
| **How often to report:** | Monthly | | | | |
| **How to review for data quality:** | Periodic review of quality of data contained within the data aggregation system should be done. | | | | |
| **How to calculate annual total:** | Annual rate will be the average of reported percentages. | | | | |
| **Data Elements (Components of indicator)** | ***Numerator (Required)****:*  Number of EMR-based reports submitted. | **Disaggregate**  **Groups**  Report type  **(Required)** | | **Disaggregates** | **Description of**  **Disaggregate**   - This disaggregation helps to determine timeliness of various types of reports |
|  |  |  | |  |  |
|  | **Denominator *(Required)***  Total number of EMR-based reports or sub-reports | **Disaggregate**  **Groups** | | **Disaggregates** | **Description of**  **Disaggregate** |
|  |  | **Disaggregate**  **Groups** | |  |  |

| **INDICATOR NAME: REPORT TIMELINESS** | | | | | |
| --- | --- | --- | --- | --- | --- |
| **Description:** | Percentage of expected EMR-based reports or sub-reports that are submitted on time. | | | | |
| **Numerator:** | Number of EMR-based reports or sub-reports submitted on time | | Country SOP should define: (a) the reports or sub-reports that are generated from data contained in the EMR and (b) a set number of days after the end of the reporting period when reports or sub-reports are supposed to have been submitted (e.g. by 5th of each month). | | |
| **Denominator** | Expected number of EMR-based reports or sub-reports. | | This indicator only applies to reports or sub-reports for data contained within the EMR. | | |
| **How to use:** | This indicator is important in determining the availability and accessibility of data for clinical, administrative and reporting decision-making. | | | | |
| **How to collect:** | This indicator is calculated directly for each facility from the data aggregation system, such as DHIS2 - as these systems already contain these data.  Report timeliness is calculated based on a whether the reports have been completed within a set number of days after the end of the reporting period. Facilities are required to report by the county’s SOP set date (e.g. 5^th^ of every month). The number of days has to be set within the data aggregation server, thus any report submitted after the set number of days is considered late.  **Percentage timeliness (reporting rate on time)** is calculated by:  (Number of actual reports submitted on time / Expected number of reports) * 100 | | | | |
| **PEPFAR MER Considerations** | None. | | | | |
| **Reporting level** | Site level, District, Country | | | | |
| **How often to report:** | Monthly | | | | |
| **How to review for data quality:** | Periodic review of quality of data contained within the data aggregation system should be done. | | | | |
| **How to calculate annual total:** | Annual rate will be the average of reported percentages. | | | | |
| **Data Elements (Components of indicator)** | ***Numerator (Required)****:*  Number of EMR-based reports submitted on time. | **Disaggregate**  **Groups**  Report type  Report transmission modality  Timeliness timestamp  **(Required)** | | **Disaggregates** | **Description of**  **Disaggregate**   - This disaggregation helps to determine timeliness of various types of reports - Report transmission modality - include automatic transmission versus manual transmission. |
|  |  |  | |  |  |
|  | **Denominator *(Required)***  Total number of EMR-based reports | **Disaggregate**  **Groups** | | **Disaggregates** | **Description of**  **Disaggregate** |
|  |  | **Disaggregate**  **Groups** | |  |  |

**Revision after NGT Exercise:**

- Recommended to disaggregate by data transmission type and add a timestamp to the timeliness.
- This indicator should only apply for reports that purely rely on all data contained in the EMR.

| **INDICATOR NAME: REPORTING COMPLETENESS** | | | | | |
| --- | --- | --- | --- | --- | --- |
| **Description:** | Number of mandatory report elements that are submitted as part of reports. | | | | |
| **Numerator:** | Number of non-blank mandatory values in reports or sub-reports. | | Country SOP should define the reports or sub-report elements that are mandatory and generated from data contained in the EMR that have to have been submitted to the national reporting system.  The expected reports or sub-reports will likely also vary by facility. | | |
| **Denominator** | Total number of mandatory cells (data elements) in reports or sub-reports. | | This indicator only applies to reports or sub-reports for data contained within the EMR. | | |
| **How to use:** | This indicator is important in determining the availability and accessibility of data for clinical, administrative and reporting decision-making. | | | | |
| **How to collect:** | This indicator is calculated directly for each facility from the data aggregation system, such as DHIS2 as these systems already contain these date.  Completeness is calculated by counting the non–blank mandatory values in the report for a specific indicator category and dividing this by the total number of mandatory cells in the report. Completeness here refers to a situation where facilities have reported on all the data they are supposed to report on, with no missing data. (In DHIS2, this is done by extracting reports for each indicator category using the pivot table).  **Percentage** completeness is calculated by:  (Number of non-blank values in reports / Total number of cells in reports) * 100 | | | | |
| **PEPFAR MER Considerations** | None. | | | | |
| **Reporting level** | Site level, District, Country | | | | |
| **How often to report:** | Per PEPFAR reporting periods | | | | |
| **How to review for data quality:** | Periodic review of quality of data contained within the data aggregation system should be done. | | | | |
| **How to calculate annual total:** | Annual rate will be the average of reported percentages. | | | | |
| **Data Elements (Components of indicator)** | ***Numerator (Required)****:*  Number of non-blank values in reports. | **Disaggregate**  **Groups**  Report type  **(Required)** | | **Disaggregates** | **Description of**  **Disaggregate**   - This disaggregation helps to determine completeness of various types of reports |
|  |  |  | |  |  |
|  | **Denominator *(Required)***  Total number of cells in reports. | **Disaggregate**  **Groups** | | **Disaggregates** | **Description of**  **Disaggregate** |
|  |  | **Disaggregate**  **Groups** | |  |  |

**Revision after NGT Exercise:**

- Should apply to the number of mandatory report elements only.
- Mandatory values can vary by facility based on the type of report or sub-report expected.

**NEW REPORTING INDICATOR SUGGESTED DURING NGT**

| **INDICATOR NAME: REPORTING CONCORDANCE** | | | | | |
| --- | --- | --- | --- | --- | --- |
| **Description:** | The extent to which data contained in paper-derived reports compare to report data derived from the EMR. (*Indicator only applies to facilities that use paper records with the data transcribed into the EMR; Retrospective data entry & hybrid system implementation modes).* | | | | |
| **Numerator:** | Number of report elements with concordant values on paper-derived reports and EMR-derived reports. | | The country SOP should define key report elements related to HIV care and treatment and derive these from paper records and from the EMR. | | |
| **Denominator** | Total number of report elements. | |  | | |
| **How to use:** | This indicator is important in determining the accuracy of EMRs at generating report results compared to primary paper-based systems. | | | | |
| **How to collect:** | Paper-based report elements are aggregated manually from registers and other paper-based sources for reports. EMR-based reports are calculated using indicator / report queries in the system. These are then compared. | | | | |
| **PEPFAR MER Considerations** | None. | | | | |
| **Reporting level** | Site level, District, Country | | | | |
| **How often to report:** | Quarterly | | | | |
| **How to review for data quality:** | Ensure that all primary paper-sources are used to derive paper-based reports. Only focus on report elements for data that are fully entered into the EMRs. | | | | |
| **How to calculate annual total:** | Annual rate will be the average of reported percentages. | | | | |
| **Data Elements (Components of indicator)** | ***Numerator (Required)****:*  Number of report elements with concordant values on paper-derived reports and EMR-derived reports. | **Disaggregate**  **Groups**  By Report element  **(Required)** | | **Disaggregates** | **Description of**  **Disaggregate** |
|  |  |  | |  |  |
|  | **Denominator *(Required)***  Total number of report elements assessed | **Disaggregate**  **Groups** | | **Disaggregates** | **Description of**  **Disaggregate** |
|  |  | **Disaggregate**  **Groups** | |  |  |
